# Improving the efficiency of soybean breeding with high-throughput canopy phenotyping

**DOI:** 10.1186/s13007-019-0519-4

**Published:** 2019-11-19

**Authors:** Fabiana Freitas Moreira, Anthony Ahau Hearst, Keith Aric Cherkauer, Katy Martin Rainey

**Affiliations:** 1 Department of Agronomy, Purdue University, 915 West State Street, West Lafayette, IN 47907 USA; 2 Department of Agricultural and Biological Engineering, Purdue University, 225 South University Street, West Lafayette, IN 47907 USA

**Keywords:** Soybean, High-throughput phenotyping, Breeding, Canopy coverage, ACC

## Abstract

**Background:**

In the early stages of plant breeding programs high-quality phenotypes are still a constraint to improve genetic gain. New field-based high-throughput phenotyping (HTP) platforms have the capacity to rapidly assess thousands of plots in a field with high spatial and temporal resolution, with the potential to measure secondary traits correlated to yield throughout the growing season. These secondary traits may be key to select more time and most efficiently soybean lines with high yield potential. Soybean average canopy coverage (ACC), measured by unmanned aerial systems (UAS), is highly heritable, with a high genetic correlation with yield. The objective of this study was to compare the direct selection for yield with indirect selection using ACC and using ACC as a covariate in the yield prediction model (Yield|ACC) in early stages of soybean breeding. In 2015 and 2016 we grew progeny rows (PR) and collected yield and days to maturity (R8) in a typical way and canopy coverage using a UAS carrying an RGB camera. The best soybean lines were then selected with three parameters, Yield, ACC and Yield|ACC, and advanced to preliminary yield trials (PYT).

**Results:**

We found that for the PYT in 2016, after adjusting yield for R8, there was no significant difference among the mean performances of the lines selected based on ACC and Yield. In the PYT in 2017 we found that the highest yield mean was from the lines directly selected for yield, but it may be due to environmental constraints in the canopy growth. Our results indicated that PR selection using Yield|ACC selected the most top-ranking lines in advanced yield trials.

**Conclusions:**

Our findings emphasize the value of aerial HTP platforms for early stages of plant breeding. Though ACC selection did not result in the best performance lines in the second year of selections, our results indicate that ACC has a role in the effective selection of high-yielding soybean lines.

## Background

Breeders are challenged to increase the rate of genetic gain. Genetic gain in a crop breeding program can be defined as $\Delta G = {{h^{2} i\sigma_{p} } \mathord{\left/ {\vphantom {{h^{2} i\sigma_{p} } L}} \right. \kern-0pt} L}$, where $h^{2}$ is the narrow-sense heritability, $i$ is the selection intensity, $\sigma_{p}$ is the phenotypic standard deviation and $L$ is the breeding cycle time or generation [1]. This equation translates theoretical quantitative genetics into parameters that breeders can manipulate in their breeding pipelines [2]. In this context genetic gain can be increased in a number of ways, including: increasing population size to increase selection intensity, shortening the breeding cycle, ensuring suitable genetic variation in the population, and obtaining accurate estimates of the genetic values [3–5]. Phenotyping directly or indirectly influences these parameters which emphasize the need for accurate, precise, relevant and cost-effective phenotypic data [6].

Plant phenotyping has recently integrated new technology from the areas of computer science, robotics, and remote sensing, resulting in high-throughput phenotyping (HTP) [6–9]. Platforms have been developed based on high capacity for data recording and speed of data collection and processing in order to capture information on structure, physiology, development, and performance of large numbers of plants multiple times throughout the growing season [8, 10]. Compared with other platforms, imagery-based field HTP using unmanned aerial systems (UAS) has the advantage of high spatial and temporal resolution [11] and is non-destructive.

There are a number of applications of a trait that can be precisely phenotyped with an HTP platform in a breeding pipeline. Secondary traits may increase prediction accuracy in multivariate pedigree or genomic prediction models [12–14]. Alternately, traits measured with HTP can be used in selection indices or for indirect selection for yield [15]. Indirect selection may be preferable when the secondary trait is easier or less expensive to measure than yield and if it can be selected out-of-season or in earlier developmental stages or generations, accelerating decision-making steps, and consequently decreasing the breeding cycle [16, 17].

In a typical soybean breeding program, after reaching desired homozygosity, a common procedure is to select individual plants and then grow the next generation in progeny rows (PR) trials [18]. At this stage, there is usually a large number of entries but a small number of seeds, limiting the experiment to unreplicated one-row plots at one location [19]. Due to these limitations, yield measurements in PR are inaccurate and may require a large investment of resources. In this scenario, HTP has the potential to remotely measure in a nondestructive manner traits correlated to yield in early stages of development, improving data quality and reducing time or cost, or, for selection [20, 21].

Several studies have demonstrated that attaining full canopy coverage, and thus maximum light interception (LI), during vegetative and early reproductive periods is responsible for yield increases in narrow-row culture due to enhanced early growth [22–24]. As management practices change over time, more recent studies using different plant populations found that rapid establishment of canopy coverage improves the interception of seasonal solar radiation, which is the foundation for crop growth and yield [25, 26]. LI efficiency, measured as leaf area index (LAI), was significantly correlated to yield in a study comparing soybean cultivars released from 1923 to 2007 [27]. In addition, the rapid development of canopy coverage can decrease soil evaporation [28] and suppress weeds [29–31].

Purcell [32] showed that soybean LI can be measured as a function of canopy coverage from images taken from above the plot using a digital camera. In addition, soybean canopy coverage can also be effectively extracted automatically from UAS-based digital imagery [33]. Xavier et al. [33] observed that average canopy coverage (ACC) measured early season was highly heritable (h^2^ = 0.77) and had a promising genetic correlation with yield (0.87), making it a valuable trait for indirect selection of yield. In the same study, they found a large effect quantitative trait locus (QTL) on soybean chromosome 19 that resulted in an estimated increase in grain yield of 47.30 kg ha^−1^ with no increase in days to maturity (− 0.24 days). Candidate genes associated with growth, development, and light responses were found in genome-wide association analysis of imagery-based canopy coverage during vegetative development [34]. Jarquin et al. [12] found that early season canopy coverage, used to calibrate genomic prediction models, improved the predictive ability for yield, suggesting that it is a valuable trait to assist selection of high yield potential lines. Thus, early season canopy coverage has the potential to be used as a secondary trait for indirect selection for yield or as covariables to improve yield estimations in quantitative genetic models [21].

While several studies have shown the value of UAS to phenotype various traits for a number of crops [35–40], to our knowledge there is no study showing the use of UAS-derived phenotypes for applied breeding purposes. In addition, no empirical studies have reported on the efficacy of using canopy coverage phenotypes in a soybean breeding pipeline. Selection experiments are useful for comparing breeding methods by enabling the assessment of realized gains of different selection categories to identify the most effective method. Our aim was to perform a selection experiment to compare the yield performance of soybean lines selected from PR based on yield with those selected based on ACC from imagery acquired with UAS.

## Methods

### Description of breeding populations

This study used 2015 and 2016 F_4:5_ progeny rows (PR) populations from the soybean breeding program at Purdue University. These trials were grown under a modified augmented design with replicated checks at the Purdue University Agronomy Center for Research and Education (ACRE) (40° 28′ 20.5″ N 86° 59′ 32.3″ W). Experimental units consisted of a one-row plot of size 1.83 m with 0.76 m row spacing and were planted on May 25, 2015, and May 24, 2016 (orientated South-North). In the 2015 PR experiment, we had 3311 plots with 2747 progenies and in 2016 PR we had 4220 plots with 4052 progenies. There was no overlap among the experimental lines in 2015 and 2016.

For both years, we advanced selected lines in early- and late-maturing preliminary yield trials (PYT early and PYT late) comprised of lines classified as earlier or later than the check IA3023. The lines selected from 2015 PR were advanced as 2016 PYT early and PYT late and the lines selected from 2016 PR were advanced as 2017 PYT early and PYT late.

The PYTs were grown in two locations and with two replications using alpha-lattice designs. The experimental unit consisted of two rows plot of 2.9 m in length in 2016 and 3.4 m in length in 2017, with 0.76 m of row spacing. For both years, one of the locations was ACRE and the second location in 2016 was at the Throckmorton-Purdue Agricultural Center (TPAC) (40° 17′ 49.1″ N 86° 54′ 12.8″ W) and in 2017 was at Ag Alumni Seed (40° 15′ 41.3″ N 86° 53′ 19.1″ W), both in Romney, IN.

Lines selected from 2016 PYT and 2017 PYT were evaluated in an advanced yield trial (AYT) in 2017 and 2018, respectively. Both trials were grown in an alpha-lattice design in two locations with either three or four replications per location. The locations were the same as described for PYT 2017. AYT plots consisted of four rows of 3.4 m length and 0.76 m spacing among rows. AYT lines were classified as early and late in the same manner as PYT.

### Phenotypic data

For all trials, grain yield and days to maturity (R8) were collected for every plot. Grain yield (g/plot) was converted to kg ha^−1^ using harvest-timed seed moisture to adjust all plot values to 13% seed moisture. R8 was expressed as days after planting when 50% of the plants in a plot had 95% of their pods mature [41].

**Fig. 1:**
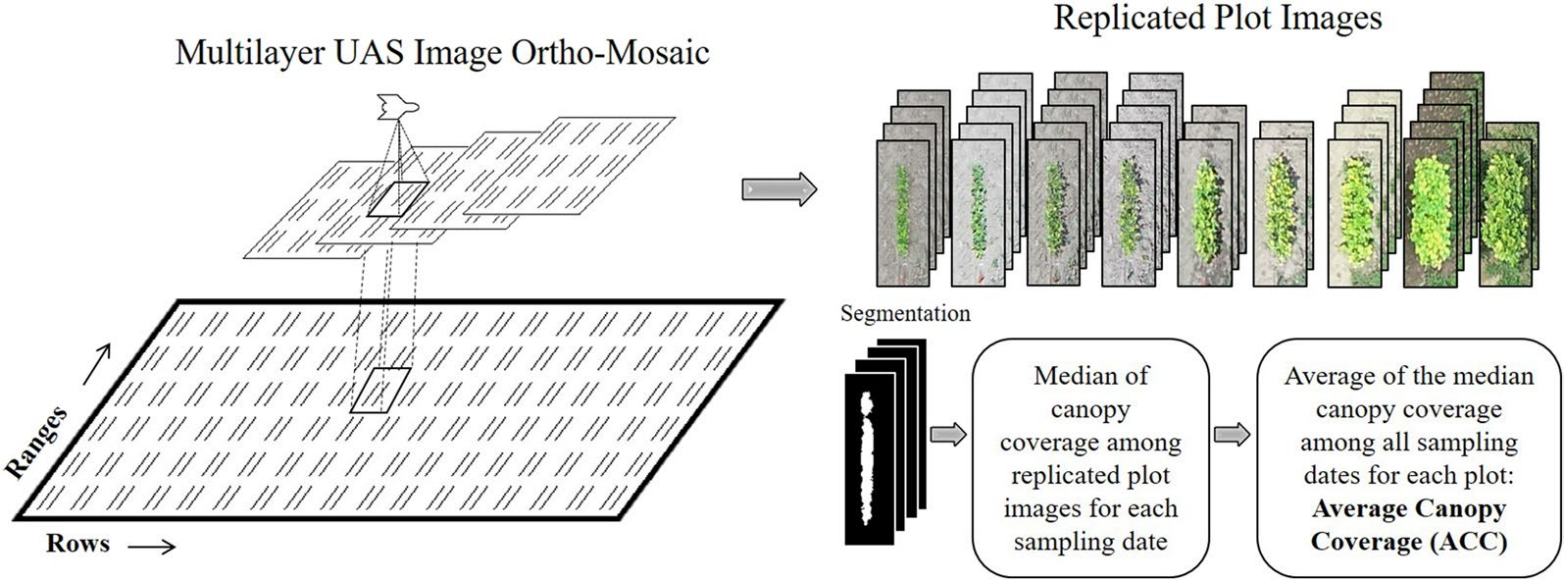
Overview of data collection and processing to acquire average canopy coverage (ACC) phenotypes

For PR 2015 and 2016 we quantified canopy coverage from aerial images collected using a fixed-wing Precision Hawk Lancaster Mark-III UAS equipped with a 14-megapixel RGB Nikon 1-J3 digital camera. Flights were performed at an altitude of 50 m, which resulted in a spatial resolution of 1.5 cm per pixel. We used eight sampling dates of early-season canopy development, ranging from 15 to 54 DAP (15, 29, 34, 37, 44, 47, 51, 54 DAP) in 2015 PR, and seven sampling dates, ranging from 20 to 56 DAP (20, 27, 31, 37, 42, 52, 56 DAP) in 2016 PR. The trials were maintained free of weeds to ensure that the images captured only soybean canopy. Image analysis, plot extraction, and classification were performed using a multilayer mosaic methodology described by Hearst [42]. This methodology allows for the extraction of the plots from ortho-rectified RGB images using map coordinates, resulting in several plot images of different perspectives from the same sampling date due to overlapping frame photos. The number of plot images from the same date varies from plot to plot. Image segmentation was done using Excess Green Index (ExG) and Otsu thresholding [42] to separated canopy vegetation from the background. Canopy coverage was calculated as the percentage of image pixels classified as canopy pixels. Median of canopy coverage values from replicated plot images was calculated for each sampling date. For each plot, average canopy coverage (ACC) was obtained by averaging the median canopy coverage among sampling dates. Figure 1 summarizes the process from image acquisition to the calculation of ACC.

### Statistical data analysis and selection methods of PR

PR 2015 and 2016 yield, R8, and ACC phenotypes were fitted in a pedigree-based mixed model to estimate variance components and breeding values, using Gibbs sampling implemented in the R package NAM [43], described as:

1 $$y_{i} = \mu + g_{i} + e_{i}$$

where $y_{i}$ is the phenotype, µ is the mean, $g_{i}$ (*i *= 1,…, number of genotypes) is the random genotype effect with $g_{i } \sim N\left( {0,\varvec{A}\sigma_{a}^{2} } \right)$ where **A** is the relationship matrix calculated using pedigrees that traced back to PR founders and $\sigma_{a}^{2}$ is the additive genetic variance, $e_{i}$ is the residual term with $e_{i} \sim {\text{N}}(0,{\mathbf{R}}\upsigma_{\text{e}}^{2}$) where **R** is a field correlation matrix considered to account for spatial variation in the field calculated as the average phenotypic value of neighbor plots [44] and $\upsigma_{\text{e}}^{2}$ is the residual variance. For yield, an additional model was fitted in order to adjust for ACC (Yield|ACC), where the fixed ACC effect (aka covariate), $\beta_{i}$ (*i *= 1,…, number of genotypes), was added to the previous model. Yield|ACC is considered a different trait than yield. The solutions for $g_{i}$ for each trait here are defined as best linear unbiased predictors (BLUP).

To estimate phenotypic correlations, we calculated Pearson’s correlations among BLUPs for the different traits. Narrow-sense heritability ($h^{2}$) was calculated using the formula:

2 $$h^{2} = \frac{{\sigma_{a}^{2} }}{{\sigma_{a}^{2} + \sigma_{e}^{2} }}$$

where $\sigma_{a}^{2}$ and $\upsigma_{\text{e}}^{2}$ are described previously.

**Fig. 2:**
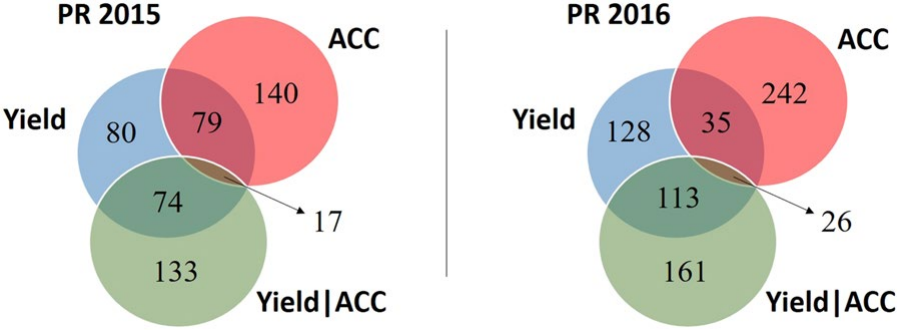
Number of lines selected from progeny rows (PR) 2015 and 2016 by each selection category

For the selection experiment, the selection categories or traits used in this study were yield BLUPs, as the traditional selection method, ACC BLUPs, and Yield|ACC BLUPs. Lines were selected based on BLUPs rankings within each selection category. For PR 2015 we selected approximately 9% of progenies for each selection category. Since some lines were selected by more than one selection category, the total lines selected was 523. In 2016, since we had more progeny lines, we decreased the selection to 7.5%. Due to the overlap of lines selected among the selection categories, we selected 705 lines. There was some deviation from the intended selection intensities due to seed limitations, field space, or logistics in the breeding pipeline. Figure 2 shows the summary of lines selected by each selection category for PR 2015 and 2016. As described above, selected lines were divided into early and late PYT.

### Evaluation of PYT and AYT

To evaluate PYT line performance, yield and R8 phenotypes across locations were fitted using restricted maximum likelihood (REML) approach, implemented in the R package lme4 [45]:

3 $$y_{ijkl} = \mu + g_{i} + loc_{j} + r_{k\left( j \right)} + b_{{l\left( {k\left( j \right)} \right)}} + (g*loc)_{ij} + e_{ijkl}$$

where $y_{ijkl}$ is the phenotype, µ is the mean, $g_{i}$ (i = 1,…, number of genotypes) is the random genotype effect with $g_{i } \sim N\left( {0,\sigma_{g}^{2} } \right)$ where $\sigma_{g}^{2}$ is the genetic variance, $loc_{j}$ (j = 1,…, number of environments) is the random location effect with $loc_{j } \sim N\left( {0,\sigma_{loc}^{2} } \right)$ where $\sigma_{loc}^{2}$ is the location variance, $r_{k\left( j \right)}$ is the random effect of *k*th replication nested within *j*th location with $r_{k\left( j \right)} \sim N\left( {0,\sigma_{r}^{2} } \right)$ where $\sigma_{r}^{2}$ is the replication within location variance, $b_{{l\left( {k\left( j \right)} \right)}}$ is the random effect of the *l*th incomplete block nested within the *k*th replication and *j*th location with $b_{{l\left( {k\left( j \right)} \right)}} \sim N\left( {0,\sigma_{b}^{2} } \right)$ where $\sigma_{b}^{2}$ is the block variance, $({\text{g*}}env)_{ij}$ is the random genotype by location interaction effect with $\left( {{\text{g}}*loc} \right)_{ij} \sim N\left( {0,\sigma_{gxloc}^{2} } \right)$. where $\sigma_{gxloc}^{2}$ e is the genotype by location variance, and $e_{ijkl}$ is the residual term with $e_{ijkl} \sim {\text{N}}\left( {0,\upsigma_{\text{e}}^{2} } \right)$ where $\upsigma_{\text{e}}^{2}$ is the residual variance. Adjusted values for yield and R8 were calculated as $\mu + g_{i}$, to express the phenotypes with units. Maturity is a confounding factor that influences yield, which may lead to misinterpretation of the yield potential of a line; therefore, we also calculated yield adjusted to R8 including R8 as a covariate in Eq. 3.

In a breeding program, the method that increases the population mean the most from one generation to the next is the preferred method; therefore, to evaluate the performance of the lines in the selected classes we performed two-sample *t*-tests to compare the adjusted yield means of lines in each selected class. The best selection category is the one producing the highest yield mean within an early or late trial, considering that all lines came from the same original populations.

Although AYT was not part of the selection experiment, we wanted to evaluate how the top-ranked lines were selected. Lines were selected from PYT using rankings of yield BLUPs and advanced to AYT. For AYT data summary Eq. 3 was used with the change of genotype to fixed effect. AYT lines were classified as early and late from R8 phenotypes.

## Results

### PR

Table 1 shows the estimated narrow-sense heritability and phenotypic Pearson’s correlations for yield, ACC, Yield|ACC, and R8 for 2015 and 2016 PR. Positive correlations were observed among all traits with Yield, with the highest observed with Yield|ACC. ACC showed low (0.01) or negative (− 0.1) correlation with R8 and negative correlation with Yield|ACC in both years. R8 and Yield|ACC were positively correlated. Narrow-sense heritability for Yield|ACC and R8 was higher than for Yield in both years. Narrow-sense heritabilities were low for ACC and Yield, but the heritability of ACC was higher than yield in 2017.

**Table 1:** Pearson’s correlations for PR 2015 (above diagonal) and 2016 (bellow diagonal) and narrow-sense heritability

*r*	Yield	ACC	Yield|ACC	R8
Yield	–	0.51	0.70	0.61
ACC	0.06	–	− 0.14	0.01
Yield|ACC	0.75	− 0.20	–	0.69
R8	0.30	− 0.10	0.20	–
*h*^2^				
PR 2015	0.23	0.06	0.35	0.36
PR 2016	0.11	0.18	0.48	0.17

Yield (kg/ha), average canopy coverage (ACC), yield given ACC (Yield|ACC) and R8 (days to maturity), progeny rows (PR)

*r* Person’s correlation, *h*^2^ narrow-sense heritability

### PYT selection category performance

**Fig. 3:**
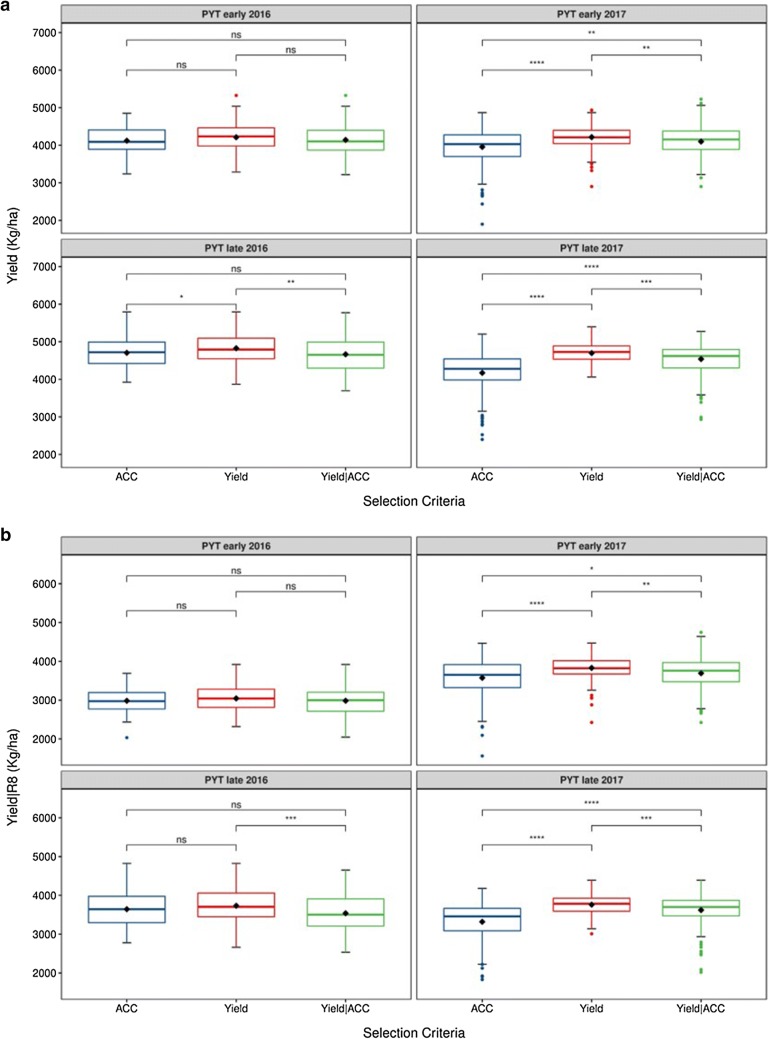
**a** Box plot of adjusted yield (Kg/ha) and **b** adjusted yield given R8 (Yield|R8) distribution for lines selected by each selection categories (Yield, ACC and Yield|ACC) for preliminary yield trials (PYT) early and late in 2016 and 2017. Diamond indicates mean for each selection categories. The line crossing the box plots are representing the median for each class. No significative (ns); p > 0.05; *p ≤ 0.05; **p ≤ 0.01; ***p ≤ 0.001; ****p ≤ 0.0001

The box plots presented in Fig. 3a show the distributions of adjusted yield values for lines in each selected class and adjusted R8 means are summarized in Additional file 1: Table S1. For PYT early 2016 the yield mean was not significantly different among the lines from different selected classes. For PYT late 2016 the lines selected by Yield had a statistically significantly higher mean yield, and there were no statistically significant differences in mean yield among the lines selected by ACC and Yield|ACC. The mean yield of the lines selected by ACC and Yield was not statistically significantly different in PYT late 2016 when considering yield adjusted by R8 (Fig. 3b). For PYT early and late in 2017, the mean yield among lines from different selected classes was statistically significantly different, and the lines selected by Yield had a higher mean yield.

### AYT yield performance

Table 2 summarizes the ten top-ranked lines in AYT 2017 and 2018. In both years, the lines were mostly selected by two selection categories. None of the ten top-ranked lines in the AYT early 2017 were selected by Yield alone in the PR stage. In the AYT late 2017 only one line was selected by Yield alone in the PR stage, in rank position ten. In AYT 2018 early and late the Yield selection category alone selected just three and two of the ten top-ranked lines, respectively. Considering both years, the number of top-ranked lines selected using only ACC and/or Yield|ACC was greater (14 lines) than the lines selected by Yield alone (6 lines).

**Table 2:** Progeny row selection categories choosing the ten top-ranked lines for advanced yield trials (AYT)

AYT early 2017	AYT late 2017	Rank	AYT early 2018	AYT late 2018
Yield, Yield|ACC	Yield|ACC	1	Yield	Yield, Yield|ACC
Yield, Yield|ACC	Yield|ACC	2	Yield, Yield|ACC	Yield|ACC
ACC, Yield	ACC, Yield|ACC	3	Yield	Yield, Yield|ACC
Yield, Yield|ACC	ACC	4	Yield, Yield|ACC	Yield|ACC
ACC, Yield	Yield|ACC	5	Yield	Yield
Yield|ACC	ACC, Yield, Yield|ACC	6	ACC, Yield	Yield, Yield|ACC
Yield, Yield|ACC	ACC, Yield, Yield|ACC	7	ACC	Yield, Yield|ACC
Yield, Yield|ACC	Yield|ACC	8	Yield, Yield|ACC	Yield
ACC, Yield, Yield|ACC	ACC, Yield|ACC	9	Yield, Yield|ACC	Yield|ACC
Yield, Yield|ACC	Yield	10	Yield|ACC	Yield|ACC

Average canopy coverage (ACC), yield given ACC (Yield|ACC)

## Discussion

The positive phenotypic correlation found in this study among yield and ACC in PR 2015 (Table 1) is in agreement with other studies [12, 33, 34]; however, this result was not repeated in PR 2016. Phenotypic correlation depends on genetic and environmental correlations, thus even when no phenotypic correlation can be estimated the traits may still be correlated genetically and environmentally [1]. Considering that some studies showed a strong positive genetic correlation between ACC and yield, the lack of phenotypic correlation in PR 2016 may be the reflection of the genetic and environmental correlations acting in opposite directions between the two traits, as well as the interaction between genotype and environment [1, 33, 46, 47].

We observed none to negative phenotypic correlations between ACC and R8 in PR 2015 and PR 2016, respectively, indicating that selection on ACC should not lead to indirect increases in maturity. In both years, ACC and Yield|ACC were negatively correlated, which is expected since adjusting yield for ACC will correct the yield data to a baseline value of ACC, thus, simplistically, yield decreases for higher ACC and increases for lower ACC.

For PR 2015 and 2016 ACC heritabilities (Table 1) were lower when compared with other studies [33, 47], but these studies used multiple environments of replicated data, and we observed comparatively lower yield and R8 heritabilities as well. Generally, low heritabilities in PR trials are expected given unreplicated single row plot trials leading to challenges in the estimation of the genetic parameters of the tested lines.

It is generally accepted that maturity confounds yield estimates in soybeans and later maturing cultivars will generally out-yield earlier maturating cultivars. In soybean breeding, yield phenotypes are sometimes corrected for R8 to better estimate yield potential per se and avoid indirect selection for later maturity. In our study, PYT early 2016 was the best scenario to compare the selection categories due to the lack of statistically significant differences in R8 among the selected classes (Additional file 1, Fig. S1). For this trial, the mean yield among the selection categories was not significantly different (Fig. 3), indicating that indirect section for yield based on ACC or using Yield|ACC would result in the same yield gain than direct selection on yield, considering that they derived from the same base population. Using ACC as a selection criterion in early stages of soybean breeding pipelines would provide advantages not only in the reduction of the time for selection but also in the cost associated with the trait measurement.

For the other three trials, PYT late 2016 and PYT 2017, there were differences in the mean R8 between at least among two of the selection categories (Additional file 1, Fig. S1). Therefore, differences in the mean yield among the selection categories may be associated with the differences in days to maturity. The yield correction for R8 changed the comparison among the selection categories Yield and ACC in PYT 2016 late, making them similarly efficient for selection (Fig. 3). Although ACC selection did not produce higher gains than Yield selection, both PYT in 2016 confirm findings from Xavier et al. [33] that assuming identical selection intensities indirect selection for yield using ACC would have a relative efficiency for selection comparable to yield direct selection. In general, the findings from PYT 2016 did not hold in 2017 trials (Fig. 3). Even after adjusting for R8 the lines selected by Yield had a higher performance than the lines selected by the other selection categories; however, the differences among the yield mean from lines selected by Yield and Yield|ACC was small for both early (~ 120 kg/ha) and late (~ 150 kg/ha) trials (Additional file 1: Table S1), which may indicate that Yield|ACC is a valuable trait for selection.

This contrasting results in trait selection efficacy observed in 2016 and 2017 may be explained by differences in canopy coverage development in PR 2015 and PR 2016, as showed in the comparison of canopy coverage development over time of the common checks among years (Additional file 1, Fig. S2). In 2015 at around 53 days after planting (DAP) we observed an average of canopy coverage of 35% in the checks, while at the same DAP in 2016 the checks had an average of almost 80% canopy coverage. This abnormal growth in 2016 produced tall plants and increased lodging (data not shown), which has a great effect in unreplicated single row plot trials where every genotype is competing with both neighbor rows. Considering that taller and bigger plants do not result in higher yields when ranking the top BLUPs, several lines that were selected based on ACC may have had poor yield potential. In addition, the lack of correlation of yield and ACC in PR 2016 may have been a result of this unusual canopy growth. Therefore, despite the evidence that one trait can be used to indirect select for yield, the breeder needs to consider the environmental influence on the trait phenotypes at the time of selection. In our case, we could have used a threshold for ACC before doing the selections, avoiding the very high values of canopy coverage, or restricted selection dates to earlier points in development.

If we consider the top 40 lines from AYT in 2017 and 2018, direct selection for yield alone selected only 6 lines from the PR trials, compared to 14 lines selected using ACC and/or Yield|ACC. Thus, despite the difference in mean performance among the selection categories in the PYT stage, we have demonstrated that ACC alone or combined with yield (Yield|ACC) are valuable secondary traits for selection in the PR stage. Yield|ACC had the best selection result in the top 10 lines for the AYT. Poor yield measurements due to harvesting errors, weather, and plot damage, lead to inaccurate representations of yield potential. Adjusting yield for early season ACC compensates for these inadequacies and is a better predictor of the real yield potential. This is in agreement with Jarquin et al. [12] results showing that early season canopy coverage increased the predictive accuracy of yield in genomic predictions models. Additionally, digital canopy coverage has a one to one relationship to LI, which in turn is an important factor for yield potential equation [32, 33, 48]. Therefore, up to a certain point, increases in LI, through ACC, will result in increases in yield when the other parameters in the yield equation are kept the same.

In this study, we have shown that the efficiency of selecting high yielding soybean lines can be improved by taking advantage of an HTP trait. Field-based HTP using UAS is robust, simple, and cost-effective and can measure a wide range of phenotypes that can be converted into useful secondary traits [2, 49]. Breeding teams need to evaluate carefully the value of these secondary traits in increasing genetic gain either in a phenotypic selection or as part of pedigree or genomic prediction schemes [2, 14]. In addition, we recommend testing different scenarios to ensure if the greater response is using the secondary trait alone or in combination with yield. However, if not in the literature, an investigation of heritability and genetic correlation to yield should be carried out to evaluate the potential of the trait.

## Conclusions

One of the most important tasks of a plant breeder is to find among the available selection criteria a combination that can promote the desirable genetic gain for the traits of interest within their breeding program. Field HTP must be integrated into a wider context in breeding programs than trait estimation, evaluation of platforms, and genetic association studies. We examined three different ways to select soybean lines from PR trials: Yield, ACC and Yield|ACC. We compared their performance in advancing selected lines in the following generations common in a soybean breeding program. We have demonstrated that the secondary trait ACC measured using an aerial HTP platform can be used for selection, alone or in combination with yield, in early stages of soybean breeding pipelines. This method may offer even more advantages when yield is low quality or can’t be phenotyped due to the high cost or extreme weather events. Further studies are needed to assess environmental effects on canopy coverage phenotypic variation in order to have optimized recommendations on the use of ACC for selecting high yielding lines in different scenarios.

## Supplementary information

**Additional file 1: Table S1.** Adjusted mean and standard deviation for yield (Kg/ha) and R8 (days to maturity) by selection criteria for preliminary yield trials (PYT) early and late in 2016 and 2017. **Figure S1.** Box plot of adjusted R8 (days to maturity) distribution for lines selected by each selection categories (Yield, ACC and Yield|ACC) for preliminary yield trials (PYT) early and late in 2016 and 2017. Diamond indicates mean for each selection categories. The line crossing the box plots are representing the median for each class. No significative (ns); p > 0.05; *p ≤ 0.05; **p ≤ 0.01; ***p ≤ 0.001; ****p ≤ 0.0001. **Figure S2.** Distribution of average canopy coverage of the checks by days after planting for progeny rows 2015 and 2016.

## Data Availability

The datasets generated and analyzed during the current study are not publicly available as they are part of the Purdue Soybean Breeding program but are available from the corresponding author on reasonable request.

## Abbreviations

ACC: average canopy coverage
AYT: advanced yield trial
BLUP: best linear unbiased predictor
DAP: days after planting
HTP: high-throughput phenotyping
LAI: leaf area index
PR: progeny rows
LI: light interception
PYT: preliminary yield trial
RGB: red, green, blue
UAS: unmanned aerial systems
