# Supplementary material for: Improving the efficiency of soybean breeding with high-throughput canopy phenotyping
Source: Plant Methods. 2019 Nov 19;15:139. doi: 10.1186/s13007-019-0519-4 (PMC6862841; doi:10.1186/s13007-019-0519-4)
Supplement: Supplementary file 1 — Additional file 1: Table S1. Adjusted mean and standard deviation for yield (Kg/ha) and R8 (days to maturity) by selection criteria for preliminary yield trials (PYT) early and late in 2016 and 2017. Figure S1. Box plot of adjusted R8 (days to maturity) distribution for lines selected by each selection categories (Yield, ACC and Yield|ACC) for preliminary yield trials (PYT) early and late in 2016 and 2017. Diamond indicates mean for each selection categories. The line crossing the box plots are representing the median for each class. No significative (ns); p > 0.05; *p ≤ 0.05; **p ≤ 0.01; ***p ≤ 0.001; ****p ≤ 0.0001. Figure S2. Distribution of average canopy coverage of the checks by days after planting for progeny rows 2015 and 2016. [file 13007_2019_519_MOESM1_ESM.docx]

**Additional file 1: Table S1:** Adjusted mean and standard deviation for yield (Kg/ha) and R8 (days to maturity) by selection criteria for preliminary yield trials (PYT) early and late in 2016 and 2017.

|  |  | ------- PYT 2016 ------- | | ------- PYT 2017 ------- | |
| --- | --- | --- | --- | --- | --- |
| Selection Criteria | Trial. | Yield | R8 | Yield | R8 |
| ACC | Early | 4121.1±361 | 117.5±3 | 3954.9±486 | 115.5±2.2 |
| Yield | Early | 4213.5±364 | 118.1±3 | 4215.5±293 | 115.7±2 |
| Yield\|ACC | Early | 4141.2±390 | 117.9±3.1 | 4099.2±422 | 117±2.9 |
| Check | Early | 3843.2±576 | 114.2±2.9 | 4310.5±512 | 113.6±2.8 |
| ACC | Late | 4704.4±397 | 127.3±4 | 4170.7±542 | 120.9±3.6 |
| Yield | Late | 4825.4±409 | 128.2±3.6 | 4697±280 | 123.8±4.1 |
| Yield\|ACC | Late | 4664.2±481 | 129.1±3.2 | 4539.4±409 | 123.2±3.1 |
| Check | Late | 4444.7±808 | 121.3±2.1 | 4397.6±311 | 113.4±2.1 |


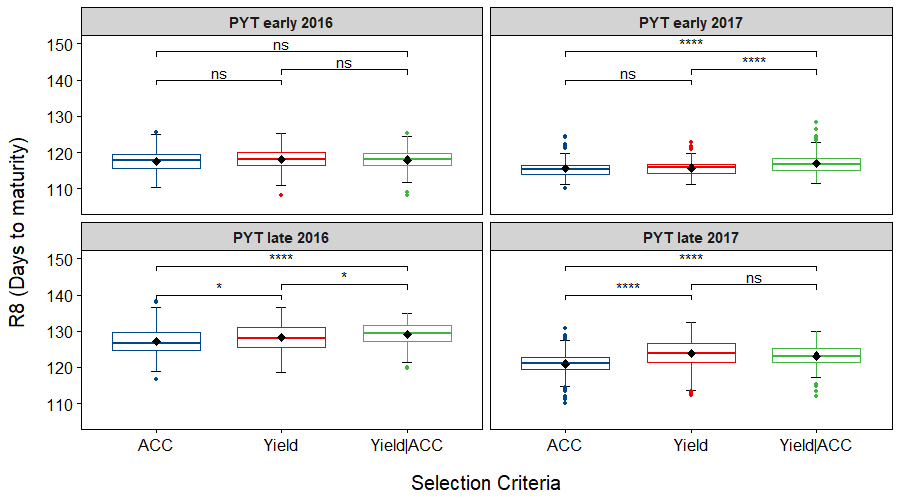


**Additional file 1: Figure S1:** Box plot of adjusted R8 (days to maturity) distribution for lines selected by each selection categories (Yield, ACC and Yield|ACC) for preliminary yield trials (PYT) early and late in 2016 and 2017. Diamond indicates mean for each selection categories. The line crossing the box plots are representing the median for each class. No significative (ns): p > 0.05; *: p <= 0.05; **: p <= 0.01; ***: p <= 0.001; ****: p <= 0.0001.


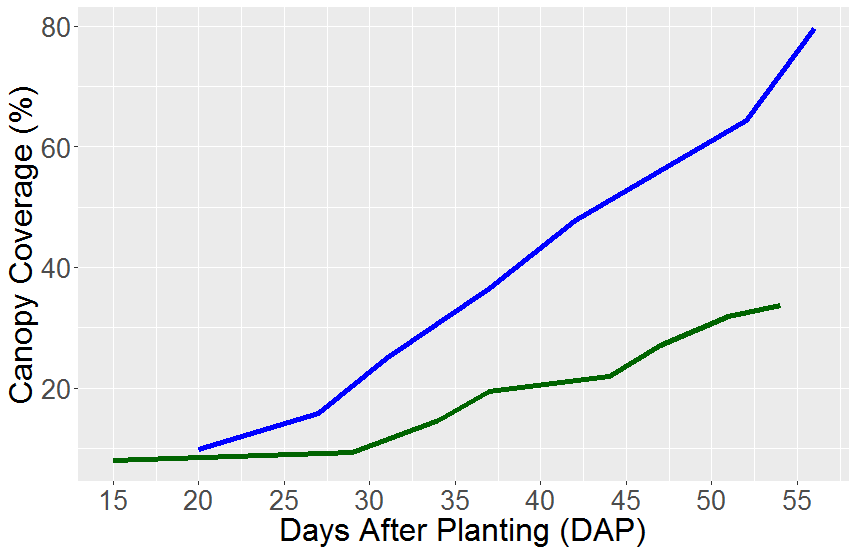

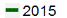

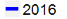


**Additional file 1: Figure S2:** Distribution of average canopy coverage of the checks by days after planting for progeny rows 2015 and 2016.
